# Mitochondria at the Crossroads of Physiology and Pathology

**DOI:** 10.3390/jcm9061971

**Published:** 2020-06-24

**Authors:** Loredana Moro

**Affiliations:** Institute of Biomembranes, Bioenergetics and Molecular Biotechnologies, National Research Council, Via Amendola 122/O, 70126 Bari, Italy; l.moro@ibiom.cnr.it

**Keywords:** mitochondria, mitochondrial dysfunction, neurodegenerative diseases, cancer, aging, inflammation, infection, cardiovascular diseases

## Abstract

Mitochondria play a crucial role in cell life and death by regulating bioenergetic and biosynthetic pathways. They are able to adapt rapidly to different microenvironmental stressors by accommodating the metabolic and biosynthetic needs of the cell. Mounting evidence places mitochondrial dysfunction at the core of several diseases, notably in the context of pathologies of the cardiovascular and central nervous system. In addition, mutations in some mitochondrial proteins are *bona fide* cancer drivers. Better understanding of the functions of these multifaceted organelles and their components may finetune our knowledge on the molecular bases of certain diseases and suggest new therapeutic avenues.

## 1. Introduction

Mitochondria are semi-autonomous organelles with a double membrane system, namely the inner and the outer mitochondrial membrane that delimit the intermembrane space. The inner mitochondrial membrane demarcates the matrix, a viscous microenvironment that contains several enzymes catalyzing a plethora of anabolic and catabolic reactions. Mitochondria contain their own genome, the mitochondrial DNA (mtDNA), a circular double-stranded DNA molecule of 16,569 bp in humans, which encodes only 13 mitochondrial proteins belonging to the electron transport chain (ETC), 22 transfer RNAs and 2 ribosomal RNAs needed to carry out the mitochondrial protein synthesis. All the other mitochondrial components are encoded by the nuclear genome. 

Mitochondria are the energy powerhouses of the cell, being responsible for 90% of energy production in the form of ATP by coupling the flux of electrons throughout the mitochondrial respiratory complexes I-IV with oxidative phosphorylation (OXPHOS). In brief, complete oxidation of nutrients through the tricarboxylic acid cycle (TCA) within mitochondria produces reduced coenzymes (NADH, FADH2) that act as electron donors. The flux of electrons through the mitochondrial respiratory chain complexes produces an electrochemical gradient used by the mitochondrial respiratory Complex V to generate ATP. Notably, the function of mitochondria in cell physiology goes beyond their role as energy producers and metabolic regulators. Indeed, these multifaceted organelles play a pivotal role in the modulation of cell death pathways and intracellular signaling [1]. The ETC is also the main cellular source of reactive oxygen species (ROS), owing to an incomplete reduction of oxygen by Complex I and Complex III. Mitochondrial ROS production can lead to oxidative damage to proteins, membranes and DNA, thus impairing the ability of mitochondria to carry out their biosynthetic and catabolic reactions, including the TCA cycle, heme synthesis, fatty acid oxidation, the urea cycle and amino acid metabolism [2]. Mitochondrial oxidative damage can also promote permeabilization of the mitochondrial outer membrane (MOMP), resulting in release of intermembrane space proteins, such as cytochrome c, and activation of the mitochondrial apoptotic pathway. Furthermore, mitochondrial ROS production promotes the opening of the mitochondrial permeability transition pore (mPTP), leading to permeabilization of the inner mitochondrial membrane to small molecules in pathological conditions, such as during ischaemia (loss of blood flow) and subsequent reperfusion [3]. 

Two mitochondria quality control mechanisms are in place to meet the functional needs of any given cell under different physiological and pathological conditions: (a) mitochondrial biogenesis, fusion and fission [4,5,6]; (b) mitophagy [7,8]. The first mechanism is a balanced process that allows maintenance of the physiological mitochondrial homeostasis when cells face metabolic or microenvironmental stresses [9]. Mitochondrial fission guarantees an adequate distribution of mitochondria in dividing cells. Mitochondrial fusion allows complementation between dysfunctional mitochondria within the cell to maximize mitochondrial performance in response to stress. Three GTPases, mitofusin 1 (Mfn1), Mfn2, and optic atrophy 1 (Opa1), are primarily involved in the regulation of mitochondrial fusion. Instead, mitochondrial fission is mainly controlled by the GTPase dynamin-related protein 1 (Drp1) [10]. Disruption of the balance between fusion and fission is associated with neurodegenerative diseases, such as Parkinson’s, and cancer [9,10]. The second mechanism, mitophagy, is a specific form of autophagy that removes damaged mitochondria and reduces the mitochondrial mass upon microenvironmental stresses, such as hypoxia and nutrient starvation, promoting cell survival [11]. Mitophagy dysregulation has been implicated in cancer development and progression [12], neurodegeneration [13] and cardiovascular diseases [7]. 

Mitochondrial dysfunction can lead to an array of diseases. Depending on the nature of the defect leading to mitochondrial dysfunction, primary and secondary mitochondrial diseases can be distinguished. Primary mitochondrial diseases develop as a consequence of germline mutations in mtDNA and/or nuclear DNA genes that encode proteins affecting mitochondrial functionality and energy production, including ETC proteins and proteins involved in mtDNA replication, such as POLG. The first primary mitochondrial disease was described in 1962 [14] and involved a 35-year-old woman displaying excessive perspiration, polyphagia, polydipsia without polyuria, asthenia and decreased body weight, symptoms that started when she was seven years old. In addition, her basal metabolic rate was +172%, and she presented with creatinuria, myopathy and pathological cardiomyogram. She was diagnosed with a disorder of the enzymatic organization of the mitochondria. Studies with mitochondria isolated from the skeletal muscle of this hypermetabolic patient revealed OXPHOS uncoupling [14]. Since then, a range of primary mitochondrial diseases has been described (reviewed in [15]). Secondary mitochondrial defects can be caused by germline mutations in genes not involved in respiration/oxidative phosphorylation or can be acquired during the lifetime upon environmental insults. Notably, environmental stress can induce mtDNA alterations leading to mitochondrial dysfunction during aging, inflammatory response, etc. [16,17]. From a pathological point of view, primary and secondary mitochondrial diseases can cause very similar symptoms, sometimes making diagnosis difficult. 

**Figure 1:**
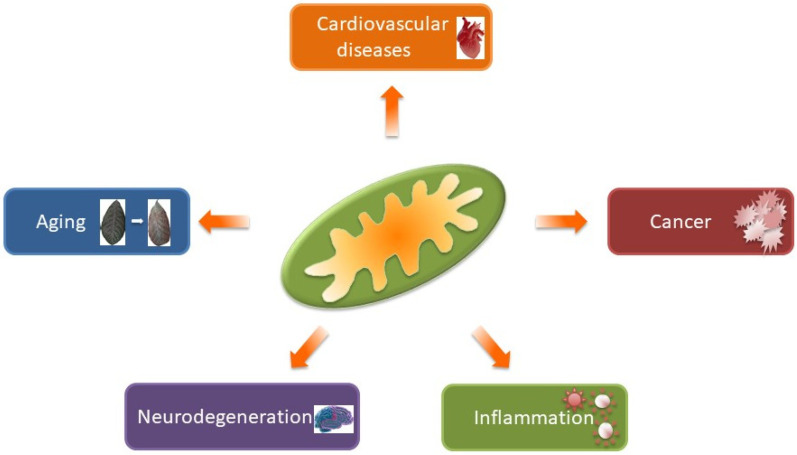
Involvement of mitochondria in different pathological conditions.

At the molecular level, mitochondrial dysfunction can affect the levels of key intracellular signaling regulators, such as ROS and Ca^2+^, that can be transmitted to the nucleus (mitochondria-to-nucleus signaling or retrograde signaling) resulting in changes in gene expression and modulation of a range of cellular functions [1,18,19,20]. In addition, the release of mtDNA and peptides from the mitochondrial matrix can activate an immune response that promotes a pro-inflammatory cascade [21]. Mitochondrial metabolites can also act as signaling molecules and epigenetic modulators. In this context, citrate, an intermediate of the TCA cycle, represents the major source of acetyl-CoA for protein acetylation, a co- and post-translational modification that regulates protein levels and intracellular signaling in physiological and pathological conditions [22]. Emerging data have also provided new evidences of connections between mitochondrial dynamics and physical contacts among mitochondria and the endoplasmic reticulum (ER), known as mitochondrial-associated ER membranes (MAMs), which can finetune the mechanisms of regulation of energy production, Ca^2+^ homeostasis, survival and apoptosis [23]. Here, a synthetic overview of the role of mitochondria in specific physiopathological conditions is provided (Figure 1).

## 2. Cardiovascular Diseases

Cardiovascular diseases are a leading cause of death worldwide. This class of diseases comprises several pathologies, including ischemic heart disease, peripheral vascular disease, cardiac arrest, heart failure, cardiomyopathies, hypertension, atherosclerosis, and arrhythmia. Mitochondria have been involved at various degrees in the pathological aspects of these diseases. Notably, mitochondrial dysfunction of muscle cells represents a key event in the prognosis of peripheral arterial disease. Reduced OXPHOS activity due to ETC impairment increases ROS levels and Ca^2+^ release from mitochondria, causing apoptosis [24]. However, if ROS levels remain below a threshold, the cells activate a defense program involving production of antioxidants and increased mitochondrial biogenesis. These mechanisms, known as mitohormesis, can limit the damage caused by repeated cycles of ischemia-reperfusion in peripheral arterial disease [24]. Pharmacological treatments that can improve mitohormesis might be a promising therapeutic approach for peripheral arterial disease and other cardiovascular diseases. Disruption of mitophagy also exacerbates the development of cardiovascular diseases [7]. Growing evidence indicates that the pharmacological targeting of the mitochondria with drugs/natural compounds able to modulate mitophagy can ameliorate cardiovascular disorders in patients and be cardioprotective [7,25]. Future studies that aim at a better understanding the pathogenesis of some cardiovascular diseases are crucial to develop mitochondria-targeting drugs in the clinic. 

## 3. Inflammation

Inflammation is a complex, protective body response to infections and tissue damage. The inflammatory response signals the immune system to repair damaged tissue and defend against pathogens (viruses, bacteria, etc.) or other harmful stimuli through secretion of specific mediators. However, when inflammation persists, it may drive various diseases and tissue damage. Mitochondrial-derived ROS play a key role in the inflammatory response. Notably, mitochondria are considered the main drivers of the NLRP3 (NOD-, LRR- and pyrin domain-containing 3) inflammasome [26,27,28,29], representing a central hub that controls innate immunity and response to inflammation. 

Among various inflammatory conditions, mitochondria are involved in the hyper-inflammatory response, also reported as cytokine storm, caused by the SARS-CoV-2 (COVID-19) respiratory infection ([30] and references therein). When macrophages and other immune cells detect viruses, they start secreting cytokines and chemokines to communicate with other immune cells [31]. Strikingly, Wuhan’s Covid-19 patients with severe clinical symptoms requiring ICU admission displayed higher levels of the cytokines/chemokines CCL2, TNF-α and CXCL10 compared to individuals with less severe symptoms [32]. The release of large quantities of pro-inflammatory cytokines and chemokines by overdriven immune effector cells sustains an aberrant systemic inflammatory response that results in the immune system attacking the body, which in turn causes the acute respiratory distress syndrome [33]. Immune cells under a hyper-inflammatory state metabolically adapt to this stress condition by favoring aerobic glycolysis over OXPHOS for energy production. This metabolic rewiring allows macrophages to become more phagocytic and favors anabolic reactions for the synthesis and secretion of cytokines and chemokines in a vicious cycle ([30] and references therein). Side by side, many biosynthetic reactions occurring in mitochondria of hyper-activated macrophages are inhibited as a consequence of OXPHOS and TCA cycle inhibition. Melatonin’s synthesis is among these reactions: acetyl-CoA, a cofactor in the rate-limiting reaction for melatonin synthesis, lacks due to the TCA cycle inhibition [30]. Thus, melatonin cannot be synthetized. Notably, melatonin is a potent anti-inflammatory and anti-oxidant and its administration to COVID-19 patients has been recently proposed as potential adjuvant treatment strategy to reduce the severity of the COVID-19 pandemic [34,35,36]. Though clinical evidences are not yet available, several scientific data supports the potential utility of melatonin to attenuate the worst symptoms of COVID-19 infection [37,38].

## 4. Aging

Mitochondrial dysfunction has long been recognized as a driver of the aging process. Early studies have linked accumulation of mitochondrial DNA mutations and the concomitant decline in ETC and OXPHOS activity to aging [29,39]. Furthermore, genetic studies in mice support a causal relation between mtDNA depletion and aging [40]. Recent evidences have confirmed that healthy centenarians retain more “intact” mtDNA copies than old people and frail centenarians [40], suggesting that “healthy” mtDNA is a hallmark of healthy aging. Besides the mtDNA status, activation of mitochondria-to-nucleus signaling pathways, particularly the mitochondrial unfolded protein response (UPR^mt^), has been implicated in aging. UPR^mt^ activation promotes transcription of several nuclear genes, such as those encoding antioxidant proteins and enzymes, which support survival, gain of the mitochondrial functionality and, thus, longevity and lifespan [41]. It should be noted that if a heteroplasmic mtDNA pool is present, UPR^mt^ activation could exacerbate mitochondrial dysfunction as it may lead to accumulation of mutant mtDNA [42]. 

Alterations in the removal of damaged mitochondria through mitophagy have also been implicated in aging. Mitophagy markedly decreases during aging in mammalian tissues and organs [43,44] and this may be responsible for the known accumulation of damaged mitochondria in aging tissues. Notably, genetic manipulations in *C. elegans* that increase mitophagy also extend the organismal lifespan [45], strengthening the connection between altered mitophagy and aging.

## 5. Neurodegeneration

Neurodegenerative diseases are characterized by changes in mitochondrial morphology and biochemical activity. Alzheimer’s (AD) and Parkinson’s (PD) disease are the most diffuse neurodegenerative illnesses among older adults. Brain cells from AD and PD patients show reduced respiratory activity and mitochondrial biogenesis [46,47]. A prominent pathological feature of AD is the impaired cerebral glucose metabolism, which is reduced by 45% in the early stages, preceding neurological impairment and atrophy, and further declines in the late stages of the disease [48]. The decrease in glucose metabolism is associated with reduced expression and activity of mitochondrial enzymes, including pyruvate dehydrogenase, isocitrate dehydrogenase and α-ketoglutarate dehydrogenase, three enzymes of the TCA cycle [49]. In addition, reduced activity of the mitochondrial respiratory complexes I, II, III and IV has also been documented [46]. Somatic mutations in the mitochondrial genome have been detected in postmortem brain tissue from AD patients, at levels higher than in healthy brains [50]. These mutations may not only affect the ETC but also trigger other neuropathological consequences, such as increased ROS production and oxidative stress in neurons and promotion of amyloidogenic processing of the amyloid precursor protein. Mitophagy is also diminished in AD’s neurons, and this may contribute to the etiopathogenesis of AD. Indeed, mitophagy was able to prevent or reverse the cognitive impairment in several AD models [51], confirming the critical involvement of mitochondria in AD.

Mutations in nuclear genes encoding mitochondrial proteins important for the proper function of mitochondria have been directly linked to PD. Notably, mutations in proteins involved in mitochondrial quality control, such as PINK1, Parkin and LRRK2, are a frequent cause of monogenic PD [52]. Loss or impaired functionality of these proteins results in mitochondrial fragmentation, dysregulation of calcium homeostasis and changes in mitochondria-endoplasmic reticulum contact sites (MERCs). Recently, mutations in Miro1, a protein important for the regulation of the structure and function of MERCs, have been causally linked to PD establishing that variants in the gene encoding for Miro1 represent rare genetic risk factors for neurodegenerative diseases like PD ([53] and references therein). 

Although there is no doubt about the involvement of mitochondrial dysfunction in AD and PD, still more research is required to identify therapeutic targets that could improve mitochondrial activity and reduce oxidative stress in neurons in the early stages of these neurodegenerative diseases. Future studies should be aimed at investigating the chronological sequence of molecular events involved in the pathogenesis of these diseases. Further investigations are also needed to assess whether mitochondrial dysfunction represents a primary cause of AD or a consequence of other molecular/genetic events.

## 6. Cancer

Mitochondrial dysfunction has been involved in different aspects of the pathogenesis of cancer, from the early steps of cancer development to cancer progression to a metastatic phenotype, and resistance to anti-cancer drugs [1,19,29]. In this context, mutations in three TCA cycle enzymes, namely succinate dehydrogenase, fumarate hydratase and isocitrate dehydrogenase, have been shown to play a causal role in carcinogenesis [54,55], thus providing compelling evidence for the involvement of mitochondrial metabolic alterations as cancer drivers. Indeed, mutations in succinate dehydrogenase predispose to hereditary paragangliomas, pheochromocytomas, neuroblastomas, gastrointestinal tumors, renal cell cancers and thyroid tumors [54]. Sporadic and hereditary mutations of fumarate hydratase trigger accumulation of an oncogenic metabolite, i.e., fumarate, that favors development of hereditary leiomyomatosis and renal cell carcinoma, Ewing sarcoma and osteosarcoma, adrenocortical carcinoma, pheochromocytoma, glioma, neuroblastoma, paraganglioma, and ependymoma [55]. Mutations in isocitrate dehydrogenase are only somatic and have been detected in about 20% of patients with acute myeloid leukemia or angioimmunoblastic T-cell lymphoma, and at lower frequencies in patients with thyroid, prostate, colorectal cancer and B-cell acute lymphoblastic leukemia [54,56]. 

Besides mutations in nuclear-encoded mitochondrial proteins, mutations in mtDNA-encoded proteins have also been implicated in the pathogenesis of cancer. The spectrum of somatic mtDNA mutations varies among different tissues, and increasing evidence shows that the load of mtDNA mutations could have prognostic value. The majority of cancer-related mtDNA mutations have been found in prostate cancer, with a total of more than 700 unique somatic mtDNA mutations associated with this cancer [57]. There is increasing evidence that mtDNA mutations/depletion may favor cancer progression to a metastatic and drug-resistant phenotype through increased production of ROS and/or activation of a mitochondria-to-nucleus signaling that leads to expression of pro-metastatic and pro-survival nuclear genes [20,29,58,59,60]. Although mtDNA damage may not be the first driver of cancer progression, it is likely that it represents a “supporter” event that facilitates and accelerates different steps of the metastatic cascade, probably within a precise time window that remains to be identified.

## 7. Conclusions

Mitochondrial dysfunction is implicated in several pathological conditions, ranging from neurodegenerative and cardiovascular diseases, to aging, cancer and inflammation. Each of these conditions shows a peculiar involvement of mitochondria. For example, up to 94% of PD patients show a defect in Miro1 function, because this protein, located on the mitochondrial surface, fails to detach from depolarized mitochondria resulting in defective mitochondrial locomotion and clearance by mitophagy [61]. These new results suggest that Miro1-based therapeutic strategies may provide new avenues to a personalized medicine for PD. 

The role of mitochondrial dysfunction in other diseases is still somehow controversial. In some cases, it may represent a driver event, like for mutations in the TCA cycle enzymes succinate dehydrogenase, fumarate hydratase and isocitrate dehydrogenase that predispose to certain types of tumors. In other cases, a transient mitochondrial dysfunction may support a metabolic rewiring needed by the cells to adapt and survive to microenvironmental stressors.
